# Development and Validation of the Needlestick Injury Prevention (N-SIP) Module

**DOI:** 10.7759/cureus.64445

**Published:** 2024-07-13

**Authors:** Ahmed Farrasyah Mohd Kutubudin, Mohd Nazri Shafei, Mohd Ismail Ibrahim, Najib Majdi Yaacob

**Affiliations:** 1 Department of Community Medicine, School of Medical Sciences, Health Campus, Universiti Sains Malaysia, Kota Bharu, MYS; 2 Department of Community Medicine, School of Medical Sciences, Health Campus, Universiti Sains Malaysia, Kota Bharu, Kelantan, MYS; 3 Department of Biostatistics and Research Methodology, School of Medical Sciences, Health Campus, Universiti Sains Malaysia, Kota Bharu, MYS

**Keywords:** house officer, nsi prevention, module validation, module development, education module

## Abstract

Introduction: Needlestick injuries (NSIs) pose a significant occupational hazard to healthcare workers (HCWs), with potential risks of exposure to bloodborne pathogens. The development of effective training modules is crucial to addressing NSI prevention and enhancing HCWs' knowledge and risk perception. This study aims to develop and validate the Needlestick Injury Prevention Module (N-SIP) using the ADDIE model (Florida State University, FL), which stands for Analysis, Design, Development, Implementation, and Evaluation, to improve NSI-related knowledge and risk perception among House Officers (HOs) in healthcare settings.

Methods: The study utilized approaches comprising literature review, module development using the ADDIE model, content validation by experts, and face validation among HOs. The N-SIP module addressed various aspects of NSI prevention, including background information, bloodborne viral infections, infection prevention practices, and occupational safety. The evaluation involved content validation by expert panels and face validation by HOs.

Results: The content validity of the N-SIP module was rigorously evaluated through expert review and validation by subject matter experts and HOs. The experts' feedback ensured the quality, relevance, and comprehensiveness of the module's instructional materials. Furthermore, face validity was assessed among HOs to ensure the module's clarity, appropriateness, and perceived effectiveness in addressing NSI prevention. The positive response from HOs indicated favorable perceptions of the module's content and instructional design, affirming its potential to effectively enhance perceptions related to NSI prevention among HCWs.

Conclusion: The development and evaluation of the N-SIP represent a significant advancement in addressing NSIs among HCWs. Through a structured approach informed by the ADDIE model, the N-SIP module offers a comprehensive and tailored learning experience aimed at enhancing NSI-related knowledge and risk perception among HOs. The study findings underscore the importance of effective training interventions in promoting a culture of safety and reducing occupational hazards in healthcare settings.

## Introduction

Needlestick injury (NSI) is a significant occupational hazard in medical settings, capable of transmitting viruses like hepatitis B virus (HBV), hepatitis C virus (HCV), and human immunodeficiency virus (HIV). Annually, about three million healthcare workers (HCWs) are exposed to bloodborne infections, with blood being the primary source of exposure in nearly all occupational illnesses.

Exposures occurred when needles contaminated with pathogens were used or when blood from infected patients came into contact with the mucous membranes of the eyes, nose, or mouth [1]. Reports from Japan indicate 40 to 50 new cases of HCV annually due to occupational accidents. The prevalence of NSIs in the workplace risks HCWs' safety and impacts patient care quality. HCWs in operating rooms, delivery rooms, emergency departments, and laboratories were at higher risk, as were those involved in cleaning, waste disposal, and handling blood-contaminated materials [2].

Nearly all countries have enacted policies to reduce NSIs, each developing its own system to address specific challenges. In Malaysia, the National Indicator Approach aims for zero new NSI cases, assessing program effectiveness, and reducing seroconversion and bloodborne transmission risks. However, despite adequate knowledge, HCWs often fail to meet the indicator due to inadequate practice of Universal Precautions (UP), as evidenced by the persistent prevalence of NSIs [3].

In 1987, the Centers for Disease Control and Prevention (CDC) recommended UP for HCWs to prevent bloodborne infections, including using gloves, goggles, and other protective gear when handling specimens or collecting blood and bodily fluids. A study indicated that adherence to UP protocols could have prevented about three-quarters of all NSI exposures among workers [4]. Therefore, raising awareness of UP guidelines and ensuring compliance was imperative to safeguard HCWs from exposure to bloodborne pathogens and the risk of infection.

The reliability of data on NSIs remains uncertain due to underreporting. The study from 1983 highlighted this issue, indicating that up to 40.0% of NSIs went unrecorded and unreported [5]. The ongoing issue of underreporting NSIs highlights a significant challenge. Low individual risk perceptions strongly influence reporting rates, leading to a high NSI incidence despite mitigation efforts. Addressing low-risk perceptions and attitudes related to underreporting may offer effective intervention strategies [5].

Given the significant and often severe consequences of NSIs and the limited effectiveness of post-exposure therapy, developing prevention techniques is crucial. Initial efforts focused on changing healthcare professionals' behavior, attitudes, and perceptions regarding NSIs. Initiatives, such as developing protective barriers, designing safer devices, substituting non-invasive treatments, and implementing administrative controls, have also been explored to prevent NSIs.

For over a decade, the CDC has recommended against recapping used needles, advocating for disposal in puncture-resistant containers unless a secure method is available. However, research shows that behavior change alone did not effectively reduce NSI incidence. Therefore, it was recognized that new prevention strategies, incorporating safer instruments, were necessary alongside behavior modification efforts [6]. Despite comprehensive guidelines for prevention, NSI risk perception remains inadequate, especially in developed nations. Over one-third of participants in a previous study admitted to recapping needles, a practice prohibited by the WHO since 1987 [7]. The study highlighted a significant flaw in the perception of NSI risk. It revealed that the underreporting of NSIs was primarily due to a misperception of the associated risks and an underestimation of the potential for bloodborne pathogen transmission from patients. This misperception is often based on self-assessment using the patient's social and medical history. This is concerning because research indicates that personal assessments of transmission risk following an NSI almost certainly underestimate the actual risk [8]. In a previous study, 20.3% of participants had a low perception of NSI risk, and this group had the highest percentage of NSIs compared to those with moderate (68.8%) and high (20.1%) risk perception levels. Furthermore, individuals with low-risk perception were more likely to experience NSIs than those with moderate and high-risk perception levels [9].

In a study conducted in Tanta, Egypt, a training module was implemented to improve knowledge and practice regarding NSIs among nurses. Initially, 93.3% of nurses had low knowledge levels, which increased to approximately 76.0% after the module. Knowledge scores rose significantly from 24.44±3.429 to 40.22±3.211 post-training (p < 0.005). Similarly, practice levels improved significantly, with 98.7% of students exhibiting poor practice before the module, which enhanced to 97.3% satisfactory levels post-module. The mean practice score showed a significant increase after training (p < 0.05). Positive correlations (p = 0.01) were found between knowledge and practice levels pre- and post-training [10].

The development and validation of the Needlestick Injury Prevention module (N-SIP) aimed to address the critical issue faced by HCWs, especially House Officers (HOs), concerning the risks associated with NSIs in hospital settings. HOs, due to their relative lack of working experience and higher frequency of performing procedures involving needles, were particularly vulnerable to NSIs. Their inexperience and high volume of needle-related tasks make them a key target group for interventions designed to mitigate NSI risks and enhance their understanding and compliance with safety protocols. Meticulously crafted, the module aimed to heighten HOs' awareness and vigilance regarding NSIs, recognizing them as potential occupational hazards within their work environment. It served as a comprehensive educational tool tailored to the specific needs of HOs, empowering them with a thorough understanding of NSIs, including causes, consequences, and preventive measures. Through evidence-based strategies and practical guidance, the module aimed to equip HOs with the necessary skills and resources to navigate NSI-related challenges effectively in their daily practice, fostering a culture of safety and risk awareness. Ultimately, it aimed to contribute to a tangible reduction in NSI occurrence and frequency among HOs, safeguarding their well-being and promoting a safer healthcare environment.

## Materials and methods

The ADDIE model (Florida State University, FL), which stands for Analysis, Design, Development, Implementation, and Evaluation, began with the analytical phase, encompassing needs assessment, issue identification, and goal establishment. Researchers explored challenges, goals, and the work environment of HCWs and HOs, alongside evaluating HOs' knowledge. A thorough literature review was conducted, scrutinizing existing content for revision, removal, and refocusing, while also assessing delivery methods for high impact. Key topics such as NSI, bloodborne viral (BBV) transmission, infection prevention, occupational safety, and accidents were systematically examined. Valuable insights were obtained on current content, best practices, resources, and optimal module delivery methods. Database searches utilized keywords like “needlestick injury” and “workplace safety” across platforms like Pubmed, Google Scholar, and Scopus, prioritizing English-language articles.

A six-member expert team was formed for the development of the N-SIP module, comprising two public health physicians, two medical officers, one infectious control nurse, and one officer from the environmental and occupational health unit. The public health physicians brought over 15 years of expertise to the team, while the medical officers had extensive experience in various medical fields, including occupational health and NSI prevention. The infectious control nurse was highly skilled in standard precautions and NSI prevention, and the environmental and occupational health unit officer was an expert leader in their field. These experts were deliberately selected to provide diverse and comprehensive input into the module's content. Ten HOs from Hospital Raja Perempuan Zainab II (HRPZ II) participated in the face validation process. The development process followed the ADDIE model, encompassing the analyze, design, develop, implement, and evaluate stages [11].

The instructional design aimed to enhance educators' teaching methods with suitable materials and methods. It provided a systematic approach to develop education and training programs consistently and reliably. This creative and interactive process was believed to improve teaching accuracy and achieve lesson objectives. By using systematically designed procedures, instructions could be made more effective, efficient, and relevant compared to less rigorously planned ones [11].

The analysis phase clarified the problem and defined objectives. During the design phase, objectives were established, activities were outlined, subject matter analyzed, lessons planned, content determined, and educational delivery methods were selected. This involved extensive discussions with stakeholders, including the Kelantan Health State Department, HRPZ II, Hospital Tanah Merah (HTM), and Hospital Sultan Ismail Petra (HSIP). The designing phase adopted a theory suited to the objectives, while the development phase constructed module contents, including lesson plans and activities.

In the development phase, theoretical plans transition into tangible instructional materials. This stage involves creating comprehensive content tailored to HCWs and HOs. Theoretical frameworks and strategies start taking shape through written materials, multimedia integration, and interactive components. The module's content validity was evaluated by six NSI experts, and face validation was conducted among HOs in HRPZ II. The content validity of N-SIP was assessed through several steps. Firstly, the researcher met with the supervisor to discuss the module and provided a soft copy for review. Feedback and comments were given in person by the supervisor, and the researcher used them to make corrections to the module. Following this, six experts were invited to evaluate the module's validity, providing remarks and criticism for improvement. Based on their feedback, modifications were made to the module. Finally, the researcher analyzed the validity of the content and reported the results. The content validity was analyzed using the item content validity index (CVI), while the face validity was assessed among HOs in HRPZ II using the face validity index (FVI). 

Content and face validity were assessed using a four-point ordinal rating system, where experts and HOs rated each chapter and topic on a scale from one to four. This study employed a widely accepted method for evaluating content validity in instrument development, which includes both Item CVI (I-CVI) and Scale-level CVI (S-CVI), as well as Item FVI (I-FVI) and Scale-level FVI (S-FVI) for face validity. The implementation and evaluation stages are not part of the current module development study but are intended for future studies to evaluate the module's effectiveness.

## Results

Module development process

**Figure 1:**
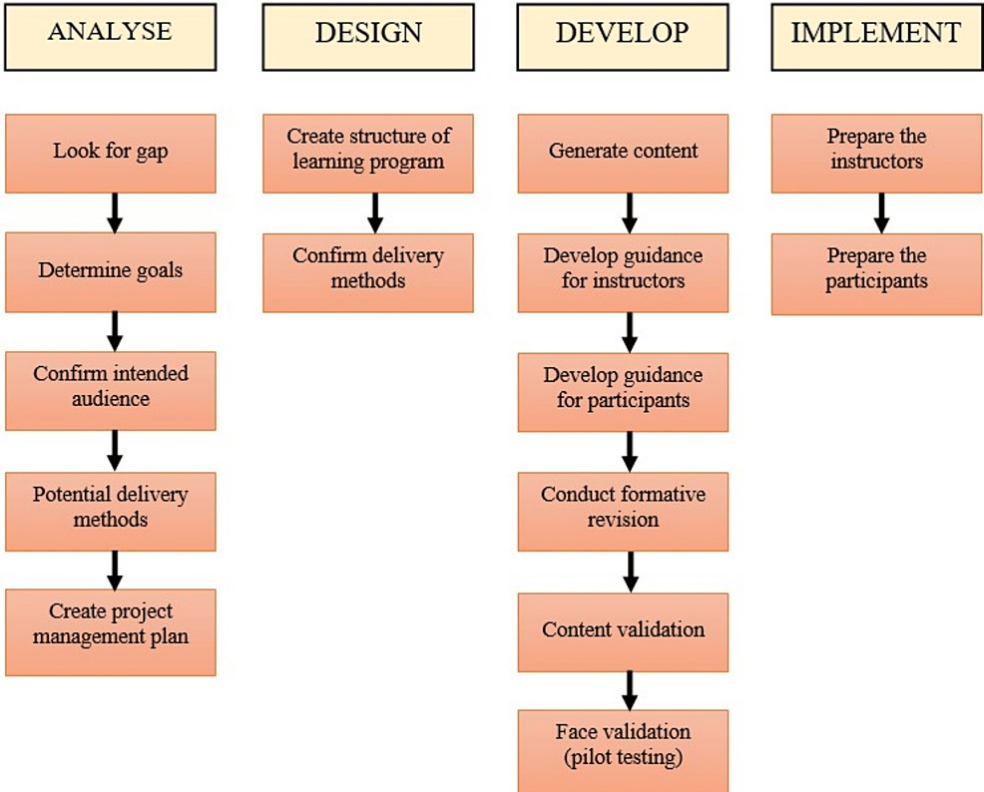
The first four stages of the ADDIE model ADDIE, Analyse Design Develop Implement Evaluation

In detail, each sub-step carried out in the first four stages is presented in Figure 1.

Analyse phase

The ideal condition expected in healthcare facilities was that all HOs receive proper training on NSI prevention, possess sufficient knowledge of standard precautions, and have a high level of risk perception. In more detail, the results of the analysis are presented in Table 1.

**Table 1:** Outcome of analyse stage HCWs, Healthcare Workers; NSI, Needlestick Injury; HOs, House Officers

Critical Questions	Answer
What are the issues/gaps with NSI prevention?	1. Inadequate Training: HCWs may not receive sufficient training on proper needle handling techniques, safe disposal practices, and the use of safety devices, leading to a higher risk of NSIs.
	2. Lack of Awareness: Some HCWs may underestimate the risks associated with NSIs or may not be aware of the proper preventive measures, resulting in a higher likelihood of accidents.
	3. Resistance to Safety Devices: Resistance from HCWs or institutions to adopt safety-engineered devices due to concerns about cost, usability, or perceived inconvenience can impede NSI prevention efforts.
	4. Inconsistent Compliance: Despite the availability of guidelines and protocols for NSI prevention, compliance with these measures may vary among HCWs and institutions, leading to gaps in prevention efforts.
	5. Work Environment Factors: Factors such as high workload, time pressure, inadequate staffing, and chaotic work environments can contribute to lapses in attention and adherence to safe practices, increasing the risk of NSIs.
	6. Reporting Barriers: Barriers to reporting NSIs, such as fear of repercussions, stigma, or lack of clear reporting mechanisms, may result in underreporting, making it difficult to identify and address the root causes of these incidents.
	7. Resource Constraints: Limited resources, including budget constraints and availability of safety equipment, may hinder healthcare institutions' ability to implement comprehensive NSI prevention programs.
Who is the focus of the problem?	HOs
Who are the target participants?	HOs
What is the desired outcome?	To increase HOs’ NSI risk perception
	Reduce the incidence of NSI
	To increase HOs' knowledge and skills regarding NSI prevention
Why is the module needed?	To address the high incidence of NSI among HOs, transmission of bloodborne pathogens, and health consequences
	To increase HOs’ knowledge and risk perception
	To prevent the incidence of NSI
How will learning occur?	The learning will comprise multiple methods, which are lecture, demonstration, roleplay, and group discussion.
What is the nature of task, knowledge, or performance?	Acquiring and applying specific competencies related to NSI prevention, including proper needle handling techniques, safe disposal practices, and adherence to institutional protocols and guidelines.

Design phase

Input from the Kelantan Health State Department highlighted the absence of a dedicated NSI prevention module at the state and national levels. The outcome of the Design Phase is presented in Table 2.

**Table 2:** Outcome of design phase HCWs, Healthcare Workers; NSI, Needlestick Injury; HOs, House Officers; N-SIP, Needlestick Injury Prevention

Aspect	Description
The focus of the Design Phase	The instructional team focused on organising the content into modules, defining learning objectives, and determining the sequence of instructional activities.
Objectives	1. Enhance awareness and understanding among HCWs, particularly HOs, regarding the risks associated with NSIs in healthcare settings.
	2. Provide comprehensive education and training on NSI prevention strategies, including safe injection practices, proper handling of sharp waste, and adherence to standard precaution.
	3. Improve HCWs' knowledge and skills in recognising, reporting, and managing NSIs to minimise their occurrence and mitigate potential health risks.
	4. Foster a culture of safety and vigilance among HCWs by promoting adherence to established protocols and best practices for NSI prevention.
	5. Equip HCWs with the necessary competencies to effectively respond to NSIs and mitigate their impact on both individual and patient safety.
	6. Facilitate the integration of NSI prevention measures into routine practice and institutional protocols within healthcare settings, thereby promoting a safer working environment for HCWs and better patient outcomes.
Basis for Design Decisions	The layout of the N-SIP module was informed by the findings of the needs assessment conducted during the prior study phase and analysis phase, which identified specific gaps in knowledge and skills related to NSI prevention among HCWs.
Forms of Design	Lectures, demonstrations, role play, and group discussion were chosen

Develop phase

Essentially, this phase brings dynamic and engaging materials to empower learners in acquiring skills to prevent NSIs effectively. Table 3 shows the chapters and topics in the N-SIP module.

**Table 3:** List of chapters and topics in N-SIP N-SIP, Needlestick Injury Prevention; HCWs, Healthcare Workers; NSI, Needlestick Injury

Chapter	Topic(s)
INTRODUCTION OF NSI	1. Background on Needlestick Injury
	2. Hazards in the Healthcare Facilities
	3. The Burden of NSI Among HCWs in Malaysia
	4. Quality Assurance (QA) Programme and National Indicator Approach
RELATIONSHIP BETWEEN BLOODBORNE VIRAL INFECTION AND NSI	5. Introduction of Bloodborne viral infection and its Management
INFECTION PREVENTION AND STANDARD PRECAUTION	6. Introduction of Infection and it’s prevention
	7. Standard Precaution
	8. Personal Protective Equipment (PPE)
PREVENTION & MANAGEMENT OF NSI	9. Risk of Getting NSI and Safe Injection Practice
	10. Prevention of NSI
	11. Reporting and Management of NSI
MANAGEMENT OF SHARP WASTE	12. Sharp Waste and Safe handling
	13. Management of Sharp Waste
OCCUPATIONAL SAFETY AND HEALTH ON ACCIDENT AND INCIDENT PREVENTION	14. Introduction of Occupational Safety and Health
	15. Risk of Occupational Injury and the Burden in Malaysia
	16. Accident and Incident and its’ Prevention

Table 4 outlined the outcomes of the development stage for the N-SIP module, which included generating content aligned with learning objectives, creating supporting media, and developing guidance materials for both HOs and instructors.

**Table 4:** Outcome of develop stage HOs, House Officers; N-SIP, Needlestick Injury Prevention; NSI, Needlestick Injury

Components	Results
Generate Content	The module was developed in alignment with the learning objectives and intended outcomes. This involved compiling information on bloodborne viral infection, infection prevention, prevention strategies, standard precaution, management of sharp waste, and occupational safety and accident.
Develop Supporting Media	Supporting media, such as presentations, videos, infographics, and interactive modules, were created to enhance the delivery of content and engage learners effectively. These media elements were designed to reinforce key concepts and facilitate comprehension and retention among HOs.
Develop Guidance for HOs	Comprehensive guidance materials were developed to provide clear instructions and support for HOs in navigating the module. This included written guidelines and instructional manuals.
Develop Guidance for instructors.	Instructors were provided with guidance materials to facilitate the effective delivery of the N-SIP module. This included training manuals, lesson plans, facilitator guides, and tips for engaging learners and addressing common challenges.
Conduct formative revision	Iterative revisions and enhancements were made to the module based on feedback from subject matter experts and pilot testing. Formative evaluations were conducted to identify areas for improvement and refine the content, media, and guidance materials to meet the needs of the target audience better.
Conduct Content Validation	Content validation was carried out by a panel of experts in the field of healthcare education and NSI prevention to assess the clarity, relevancy, and completeness of the module content, ensuring that it aligns with established guidelines and best practices.
Conduct Face Validation (Pilot Testing)	A pilot test (face validation) was conducted with a sample of HOs to evaluate the clarity and understandability of the module.

Table 5 described the prevention program and the educational materials used, focusing on lectures, demonstrations, role play, and group discussions. These methods aimed to provide comprehensive training on bloodborne viral infections, NSI prevention, infection control, and occupational safety for healthcare professionals.

**Table 5:** NSI prevention program and educational materials used N-SIP, Needlestick Injury Prevention; NSI, Needlestick Injury

Education Material	Objectives	Content	Method of Implementation
Lecture	To give an overview and deliver N-SIP to participants in an interactive way	Bloodborne viral infection, Prevention of NSI, Infection prevention, Standard Precaution, Management of Sharp waste, Occupational Safety and Accidents.	Six lecture sessions of around five hours duration by the main researcher, research team members, and invited speakers.
Demonstration	To deliver NSI prevention in an interactive way To ensure participants are able to learn correctly the steps and procedure		Conducted by two experienced infection control nurses using videos and action.
Roleplay	To ensure that participants can practice what was learned during the demonstration and apply to daily work routine		Conducted by two experienced infection control nurses using videos and action.
Group discussion	To supplement and sustain the knowledge		Conducted by the main researcher and materials including videos and graphics were given.

Table 6 provided a detailed guide for demonstration and role-play activities focused on standard precautions, personal protective equipment uses, and sharp waste management. These activities, spanning 60 minutes each, aimed to enhance practical skills and reinforce safety protocols through interactive and hands-on training sessions.

**Table 6:** Demonstration and role play guide SP, Standard Precaution; PPE, Personal Protective Equipment; HCWs, Healthcare Workers; NSI, Needlestick Injury

Activities	Duration	Materials Needed	Procedure
Standard Precaution & PPE	60 minutes	Gloves, masks, and sanitiser	Activity 1: Demonstration of standard precautions and PPE use
			Activity 2: Roleplay scenario for SP and PPE use
			Activity 3: Action to be taken post NSI
Management of Sharp Waste	60 minutes	Sharp container, kidney dish, syringes and needles, procedure trolley, wipe disinfectant, gloves, gowns, and masks	Activity 1: Demonstration of safe sharp waste disposal
			Activity 2: Roleplay scenario for sharps waste management

Module validity (content and face validation)

A scoring guide was provided to aid experts in the evaluation process (Table 7).

**Table 7:** Scoring method for content validation

Relevancy	Clarity
1[ not relevant]	1[not clear]
2[item need some revision]	2[item need some revision]
3[relevant but need minor revision]	3[clear but need minor revision]
4[ very relevant]	4[very clear]

The N-SIP received great ratings from six experts, with both I-CVI and S-CVI values assessed as 1.0. Table 8 outlined the module's relevancy and clarity. Experts praised the formatting, language, graphics, and illustrations.

**Table 8:** Content validity of N-SIP by experts (n=6) N-SIP, Needlestick Injury Prevention; NSI, Needlestick Injury; HCWs, Healthcare Workers; I-CVI, Item-level Content Validity Index; S-CVI, Scale-level Content Validity Index; Ave, Average; UA, Universal Agreement; PH, Public Health; ICN, Infectious Control Nurse; SHO, Safety Health Officer

Item	Relevancy (I-CVI)				Clarity(I-CVI)			
	PH	ICN	SHO	Overall	PH	ICN	SHO	Overall
Background of NSI	1.0	1.0	1.0	1.0	1.0	1.0	1.0	1.0
Hazards in healthcare facilities.	1.0	1.0	1.0	1.0	1.0	1.0	1.0	1.0
Burden of NSI among HCWs in Malaysia	1.0	1.0	1.0	1.0	1.0	1.0	1.0	1.0
Quality Assurance Program and National Indicator Approach	1.0	1.0	1.0	1.0	1.0	1.0	1.0	1.0
NSI and who at risk	1.0	1.0	1.0	1.0	1.0	1.0	1.0	1.0
Bloodborne viral infection	1.0	1.0	1.0	1.0	1.0	1.0	1.0	1.0
Infection and chain of infection	1.0	1.0	1.0	1.0	1.0	1.0	1.0	1.0
Standard Precaution	1.0	1.0	1.0	1.0	1.0	1.0	1.0	1.0
Hierarchy of control & Personal Protective Equipment	1.0	1.0	1.0	1.0	1.0	1.0	1.0	1.0
Safe and unsafe practice in common procedures	1.0	1.0	1.0	1.0	1.0	1.0	1.0	1.0
Standard procedures of NSI prevention	1.0	1.0	1.0	1.0	1.0	1.0	1.0	1.0
Reporting and management of NSI	1.0	1.0	1.0	1.0	1.0	1.0	1.0	1.0
Sharp waste and safe handling.	1.0	1.0	1.0	1.0	1.0	1.0	1.0	1.0
Sharp container, management and disposing of sharp waste	1.0	1.0	1.0	1.0	1.0	1.0	1.0	1.0
Occupational safety and health (OSH)	1.0	1.0	1.0	1.0	1.0	1.0	1.0	1.0
Burden and who at risk to get occupational injury.	1.0	1.0	1.0	1.0	1.0	1.0	1.0	1.0
Accident, incident, and its' prevention	1.0	1.0	1.0	1.0	1.0	1.0	1.0	1.0
Average I-CVI (S-CVI/Ave)	1.0	1.0	1.0	1.0	1.0	1.0	1.0	1.0
S-CVI (S-CVI/UA)	1.0	1.0	1.0	1.0	1.0	1.0	1.0	1.0

A scoring guide was provided to aid in the evaluation process (Table 9).

**Table 9:** Scoring method for face validation

Understandability	Clarity
1[ cannot be understood]	1[not clear]
2[quite understandable]	2[item need some revision]
3[can be understood]	3[clear but need minor revision]
4[ easy to understand]	4[very clear]

The majority of the HOs concurred that the materials were readable and informative. Additionally, they concurred on the arrangement style and thought the module materials were easy to understand and clear. Table 10 provided information on how HOs perceived the items in the N-SIP module.

**Table 10:** Face validity of N-SIP by HOs (n=10) N-SIP, Needlestick Injury Prevention; NSI, Needlestick Injury; HCWs, Healthcare Workers; I-FVI, Item-level Face Validity Index; S-FVI, Scale-level Face Validity Index; Ave, Average; UA, Universal Agreement; PH, Public Health; ICN, Infectious Control Nurse; SHO, Safety Health Officer

Item	Understandability (I-FVI)				Clarity(I-FVI)			
	PH	ICN	SHO	Overall	PH	ICN	SHO	Overall
Background of NSI	1.0	1.0	1.0	1.0	1.0	1.0	1.0	1.0
Hazards in healthcare facilities.	1.0	1.0	1.0	1.0	1.0	1.0	1.0	1.0
Burden of NSI among HCWs in Malaysia	1.0	1.0	1.0	1.0	1.0	1.0	1.0	1.0
Quality Assurance Program and National Indicator Approach	1.0	1.0	1.0	1.0	1.0	1.0	1.0	1.0
NSI and who at risk	1.0	1.0	1.0	1.0	1.0	1.0	1.0	1.0
Bloodborne viral infection	1.0	1.0	1.0	1.0	1.0	1.0	1.0	1.0
Infection and chain of infection	1.0	1.0	1.0	1.0	1.0	1.0	1.0	1.0
Standard Precaution	1.0	1.0	1.0	1.0	1.0	1.0	1.0	1.0
Hierarchy of control & Personal Protective Equipment	1.0	1.0	1.0	1.0	1.0	1.0	1.0	1.0
Safe and unsafe practice in common procedures	1.0	1.0	1.0	1.0	1.0	1.0	1.0	1.0
Standard procedures of NSI prevention	1.0	1.0	1.0	1.0	1.0	1.0	1.0	1.0
Reporting and management of NSI	1.0	1.0	1.0	1.0	1.0	1.0	1.0	1.0
Sharp waste and safe handling.	1.0	1.0	1.0	1.0	1.0	1.0	1.0	1.0
Sharp container, management and disposing of sharp waste	1.0	1.0	1.0	1.0	1.0	1.0	1.0	1.0
Occupational safety and health (OSH)	1.0	1.0	1.0	1.0	1.0	1.0	1.0	1.0
Burden and who at risk to get occupational injury.	1.0	1.0	1.0	1.0	1.0	1.0	1.0	1.0
Accident, incident, and its' prevention	1.0	1.0	1.0	1.0	1.0	1.0	1.0	1.0
Average I-FVI (S-FVI/Ave)	1.0	1.0	1.0	1.0	1.0	1.0	1.0	1.0
S-FVI (S-FVI/UA)	1.0	1.0	1.0	1.0	1.0	1.0	1.0	1.0

## Discussion

This study utilized the ADDIE model to enhance teaching and learning in education, with a specific focus on increasing HOs' knowledge and risk perception. The ADDIE model is a widely recognized instructional design framework. It was chosen for this module development due to its systematic approach that thoroughly addresses all aspects of the educational process. Previous research has demonstrated the effectiveness of the ADDIE model in improving the teaching and learning process [12-14].

The results showed that the module was applicable. This was due to the high content validity of N-SIP. For content validity with six experts, the I-CVI must be at least 83.0%, which reflects only one disagreement. This finding was consistent with a previous study, which obtained a content validity of >83.0% [15].

N-SIP had practical and theoretical considerations. The research findings demonstrate the module's high validity in enhancing HOs' knowledge and risk perception of NSIs. Healthcare educators can confidently use the module as a structured framework to effectively educate HOs on NSI prevention, increase knowledge, and heighten risk perception, fulfilling its intended objectives [16].

The N-SIP module shifts the educator's role from a traditional teacher-centered approach to a student-centered one. Educators will act as facilitators, encouraging discussions and active engagement among HOs. This approach enhances the learning experience and empowers HOs to take ownership of their learning process [17]. The N-SIP module offers educators insights into enhancing critical thinking in occupational safety. By integrating risk perception and knowledge acquisition into the curriculum, it helps nurture cautious decision-making among HOs, promoting safer healthcare practices [18].

During the development of the N-SIP, the analysis stage revealed gaps in NSI knowledge and risk perception among HOs, echoing previous studies on inadequate NSI training programs. Many HOs reported never receiving NSI education [19,20]. Factors contributing to this include the lack of a standardized NSI prevention module, insufficient resources, time constraints, low prioritization by hospital management, and some healthcare institutions perceiving NSIs as low-risk incidents [21-23].

The analysis showed that many HOs inconsistently apply NSI prevention strategies, reflecting findings of inadequate NSI prevention knowledge and skills among HCWs and HOs in Malaysia [20]. Several factors contribute to this, including learning environments that do not prioritize NSI prevention and a lack of modules focused on empowering HOs' NSI prevention skills [22].

In the design stage, efforts focused on developing NSI prevention materials to enhance HOs' knowledge and risk perception. Based on previous literature, methods such as lectures, demonstrations, role play, and group discussions were crafted for comprehensive learning and delivery of N-SIP [24,25].

Each chapter in the develop phase enhances HCWs' knowledge and risk perception of NSIs. The introduction chapter provides a foundational understanding of NSIs' prevalence, causes, and consequences, highlighting the importance of prevention. Subsequent chapters detail the relationship between BBV infections and NSIs, equipping HCWs with the essential knowledge to mitigate transmission risks. Emphasizing infection prevention and standard precaution, the module empowers HCWs to minimize NSI and BBV transmission risks in healthcare settings. The chapter on NSI prevention and management covers practical strategies to minimize risks during clinical procedures, including safe injection practices and sharps disposal. The N-SIP module provides essential guidance on handling and disposing of sharp waste, equipping HCWs with the skills to prevent NSIs and protect themselves and others. It emphasizes recognizing workplace hazards and adhering to safety protocols to mitigate NSI risks and other injuries. Each chapter was designed to enhance HCWs' knowledge and risk perception of NSIs, effectively promoting a safer healthcare work environment.

Following the development of instructional materials, rigorous validation by subject matter experts and HOs ensured the quality and relevance of the N-SIP module. Following the develop stage of the N-SIP module, the subsequent study will focus on its implementation to assess its effectiveness which will be done later on. The evaluation of HOs following the delivery of the N-SIP module is a critical step in assessing the effectiveness of the educational intervention in enhancing HOs' knowledge and risk perception of NSIs.

The strengths of this study lie in its comprehensive development of the N-SIP module using the ADDIE model, addressing key aspects of NSI prevention such as infection control, BBV infections, and occupational safety. This holistic approach ensures a structured learning experience for HCWs. The module's design is informed by a thorough literature review and gap analysis, tailoring it to meet the specific needs of HCWs and enhancing its relevance and effectiveness. Additionally, the planned future evaluation using a validated questionnaire will provide valuable insights into the module's impact on knowledge and risk perception of NSIs among HOs, reinforcing its efficacy.

However, this study has several limitations. Firstly, the generalizability of the findings may be restricted to the specific context of the healthcare setting in Malaysia and the characteristics of the HOs involved. The focus solely on HOs may overlook the perspectives and experiences of other healthcare professionals who also face the risks of NSIs. Lastly, while validation efforts were undertaken, the validation process could benefit from further refinement and expansion to ensure the robustness and applicability of the findings across different settings and populations.

## Conclusions

The N-SIP module represents a significant advancement in addressing NSIs among HCWs. Developed using the ADDIE model, the module was successfully created and tailored to equip HCWs with essential knowledge and skills to effectively recognize, prevent, and manage NSIs. The rigorous validation process, which included high content and face validation scores, underscores the module's effectiveness. These scores affirm that the module is both comprehensive and user-friendly, meeting the learning needs of HCWs.

Through collaboration with healthcare experts, educators, and frontline workers, the N-SIP module was refined to ensure its relevance and practicality across diverse healthcare settings. Integrating this module into training programs is vital for fostering a culture of safety, with continued evaluation and advocacy necessary to sustain its impact. Ultimately, the N-SIP module has the potential to significantly reduce NSIs, thereby improving safety for both HCWs and patients.
